# A differential equations model-fitting analysis of COVID-19 epidemiological data to explain multi-wave dynamics

**DOI:** 10.1038/s41598-021-95494-6

**Published:** 2021-08-11

**Authors:** Maria Jardim Beira, Pedro José Sebastião

**Affiliations:** Center of Physics and Engineering of Advanced Materials, Departamento de Física, Instituto Superior Técnico, Universidade de Lisboa, Av. Rovisco Pais, 1049-001 Lisbon, Portugal

**Keywords:** Software, Engineering, Applied mathematics

## Abstract

Compartmental epidemiological models are, by far, the most popular in the study of dynamics related with infectious diseases. It is, therefore, not surprising that they are frequently used to study the current COVID-19 pandemic. Taking advantage of the real-time availability of COVID-19 related data, we perform a compartmental model fitting analysis of the portuguese case, using an online open-access platform with the integrated capability of solving systems of differential equations. This analysis enabled the data-driven validation of the used model and was the basis for robust projections of different future scenarios, namely, increasing the detected infected population, reopening schools at different moments, allowing Easter celebrations to take place and population vaccination. The method presented in this work can easily be used to perform the non-trivial task of simultaneously fitting differential equation solutions to different epidemiological data sets, regardless of the model or country that might be considered in the analysis.

## Introduction

Back on January 2020, when the World Health Organization (WHO) first mentioned a cluster of pneumonia cases in Wuhan, no one expected that by the 11th of March, COVID-19 (COronaVIrus Disease-19) would have spread across the globe and would be declared a pandemic^1^. Infectious diseases have accompanied mankind since the beginning of its existence and are known to have profoundly influenced the fate of entire nations upon their evolution to local epidemics or to great pandemics^2^. COVID-19, caused by SARS-CoV-2 (Severe Acute Respiratory Syndrome CoronaVirus 2), is, however, the first pandemic to ever reach every country in the world within this era of global information. Furthermore, this happened despite what might be considered the largest lock-down in history.

The well-known works of W. O. Kermack and A. G. McKendrick^3^, published in 1927, on the compartmental SIR model, have paved the way for epidemiologists to mathematically describe the dynamics of infectious diseases and became increasingly popular among the scientific community, particularly for the analysis of the current pandemic^4–12^. These compartmental SIR-models are composed of systems of differential equations that, except for a few particular cases, have no analytical solutions^13^. Probably, this is the main reason why only a very limited number of scientific works actually provide the fit of the SIR-type model differential equations systems to the COVID-19 related data^6, 7, 11^ and why others prefer to fit the data using approximated analytical expressions, such as the logistic function^14, 15^.

The number of daily new cases, deceased and recovered related to COVID-19 is made available to the general public in close to real time^16, 17^, which makes it possible to test models by actually fitting the existing data. Here we propose a model based on the comprehensive PSEIRD(S) model by Beira et al.^11^ to fit the data sets related to the infected, deceased and hospitalized cases reported for Portugal, a country that had a severe post-Christmas outbreak. The PSEIRD(S) model was modified in order to account for vaccination and avoiding infectious pathways that required the use of parameters which could not be independently estimated, such as the fraction of infected individuals that, regardless of being detected, do not infect others. Still, it could be argued that this modified PSEIRD(S) model is overly complex for the description of the COVID-19 dynamics, but, as it will become evident in the following sections, it represents a balance between effective number of model parameters and an adequate description of this epidemic.

Fitting the solutions of differential equations is, undoubtedly, a non-trivial process and usually requires an extensive overhead work to implement dedicated software. In view of this fact, a specific ordinary differential equation solver was developed to extend the capabilities of the online open-access platform *fitteia.org*, originally developed for general model fitting purposes^18^ . This platform was used to test the modified PSEIRD(S) model and may be used to test other models/assumptions. It is important to note that, as it was observed for the original PSEIRD(S) model when used to analyze the data for 11 countries, the method here presented will eventually work for any country and may be used for the validation of any model.

## Methods

### Model

**Figure 1:**
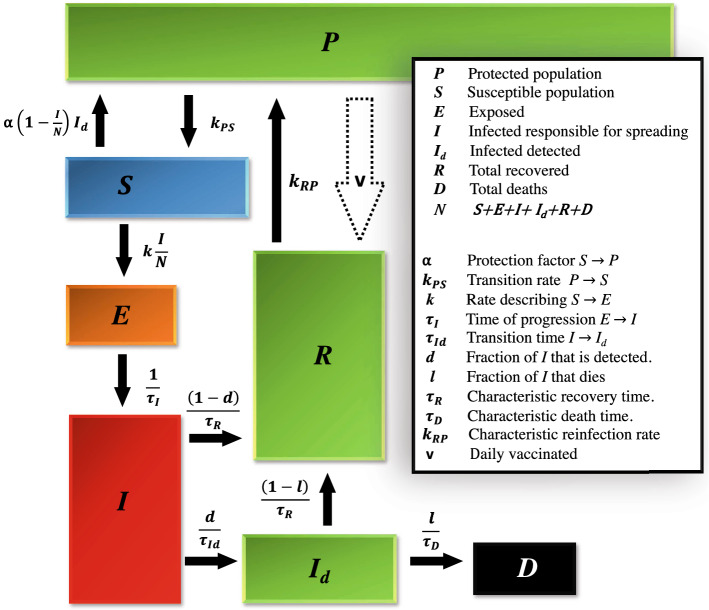
Compartment diagram of the alternative SEIRD(s) model, modified in order to accommodate protected individuals, ***P***, to discriminate between detected, ***I***$_d$, and non-detected, ***I***, infectious cases, and to include the process of vaccination. The flows between compartments are normalized and mathematically related to the model parameters, which are listed and briefly described in the box on the right hand side.

Figure 1 shows the outline of the compartmental model used in this work to explain the dynamics of COVID-19.

The model considers that, when the virus first reaches a country, the initial number of susceptible individuals is equal to the entire population of that country. As people become aware of the presence of the virus they tend to protect themselves and, as a consequence, become a part of the ***P*** compartment. The rate of this transition was considered to be proportional to the number of detected active infected and to a factor $\alpha$ that we decided to call *protection factor*. This flow happens as a consequence of individuals following sanitary and social distancing measures, either imposed by the government or self-imposed by each individual as a result of risk perception. It is generally accepted that the awareness state of the population will influence the dynamics of an infectious disease, as described in previous works^8, 19^. There may also be an involuntary flux from ***S*** to ***P*** corresponding to the part of the population that is physically or geographically isolated from the virus.

Multiple outbreaks can be explained by considering flows from ***P*** to ***S*** with rates $k_{PS}$, specific for each outbreak. These flows are a consequence of governments and/or individuals relaxing some or all of the sanitary and social distancing measures.

From the susceptible compartment, individuals evolve, upon contacting with an infectious individual from compartment ***I***, to an exposed state, ***E***, where they are infected but not yet infectious. This transition happens at a rate *k*, which is the product of the number of daily contacts and the probability of a contact generating an infection. An exposed individual evolves to ***I*** with a characteristic time, $\tau _I$, related with the incubation period of the virus. From ***I***, a fraction *d* is detected and transitions into ***I***$_d$, with characteristic time $\tau _{Id}$, while the remaining individuals recover and go into ***R***, with characteristic recovery time $\tau _R$. The differentiation between detected and undetected infectious individuals is crucial for the description of the COVID-19 dynamics, as undetected individuals largely promote the dissemiation of the disease^20^. In the PSEIRD(S) model, we assumed that detected individuals no longer propagate the disease. From compartment ***I***$_d$, a fraction *l* dies and becomes part of compartment ***D***, while the fraction $(1-l)$ recovers, with characteristic time $\tau _R$. Finally, vaccination and loss of immunity can also be accounted for by knowing the daily vaccinated, *v*, and applying the characteristic reinfection rate, $k_{RP}$, in the transition from ***R*** to ***P***.

The main difference between this modified PSEIRD(S) model and the original PSEIRD(S) model^11^ is the elimination of the infection pathway by which a fraction of the infectious individuals would not infect susceptible people. It was further assumed that the deceased came exclusively from the detected infected compartment, **I**$_d$, as opposed to what was considered for the original PSEIRD(S) model. These modifications do not disregard the importance of human action in the dynamics of COVID-19, as testing (reflected in parameters *d* and $\tau _{Id}$), risk-perception (reflected in $\alpha$) and sanitary/social distancing measures, as well as general protection (reflected in ***P***) are still considered in the modified PSEIRD(S) model. The modified PSEIRD(S) model, additionally takes the vaccination process into consideration.

The mathematical description of the seven compartments composing this model requires the following set of differential equations:

1 $$\begin{aligned} \frac{d{\varvec{P}}}{dt}& = k _{RP}{{\varvec{R}}}-k _{PS}{\varvec{P}}-v \end{aligned}$$

2 $$\begin{aligned} \frac{d{\varvec{S}}}{dt}& = k _{PS}{\varvec{P}}-k\frac{{\varvec{I}}}{{\varvec{N}}}{\varvec{S}} \end{aligned}$$

3 $$\begin{aligned} \frac{d{\varvec{E}}}{dt}& = k\frac{{\varvec{I}}}{{\varvec{N}}}{\varvec{S}}-\frac{1}{\tau _I}{\varvec{E}} \end{aligned}$$

4 $$\begin{aligned} \frac{d{\varvec{I}}}{dt}& = \frac{1}{\tau _I}{\varvec{E}}-\left[ \frac{(1-d)}{\tau _R}+\frac{d}{\tau _{Id}}\right] {\varvec{I}} \end{aligned}$$

5 $$\begin{aligned} \frac{d{\varvec{I}}_d}{dt}& = \frac{d}{\tau _{Id}}{\varvec{I}}-\left[ \frac{(1-l)}{\tau _R}+\frac{l}{\tau _D}\right] {\varvec{I}}_d \end{aligned}$$

6 $$\begin{aligned} \frac{d{\varvec{R}}}{dt}& = \frac{1}{\tau _R}\left[ (1-l){\varvec{I}}_d+(1-d){\varvec{I}}\right] +v-k _{RP}{\varvec{R}} \end{aligned}$$

7 $$\begin{aligned} \frac{d{\varvec{D}}}{dt}& = \frac{l}{\tau _D}{\varvec{I}}_d. \end{aligned}$$

In order to fit the data for the cumulative number of detected cases, $N_{T_d}$, only the flow that goes into ***I***$_d$ (see Eq. 5) needs to be considered, which leads to Eq. (8)

8 $$\begin{aligned} \frac{dN_{T_d}}{dt}=\frac{d}{\tau _{Id}}{\varvec{I}} \end{aligned}$$

The daily detected cases are obtained from the derivative of the total detected cases

9 $$\begin{aligned} \frac{dN_{I_d}}{dt}=\frac{d}{\tau _{Id}\tau _I}{\varvec{E}}-\left[ \frac{(1-d)}{\tau _R}+\frac{d}{\tau _{Id}}\right] N_{I_d}. \end{aligned}$$

Analogously, the daily deceased can be obtained from the derivative of the cumulative number of deaths, leading to Eq. (10).

10 $$\begin{aligned} \frac{dN_{D}}{dt}=\frac{l \,\, d}{\tau _{Id}\tau _D}{\varvec{I}}-\left[ \frac{(1-l)}{\tau _R}+\frac{l}{\tau _D}\right] N_{D} \end{aligned}$$

One of the main concerns of public health authorities, is to avoid the collapse of hospital services in the event of a dramatic increase of COVID-19 patients. In view of this fact, it is also of interest to follow the time evolution of the number of COVID-19 related hospitalized cases and of the number of patients in intensive care units (ICU). Both these groups are a fraction of the infected detected population, present a specific rate of hospitalization and leave this state with a specific characteristic time. The differential equations that characterize the flow into and from these compartments can be written as

11 $$\begin{aligned} \frac{dI_h}{dt}& = k_h {\varvec{I}}_d-\frac{1}{\tau _{R_h}}I_h, \end{aligned}$$

12 $$\begin{aligned} \frac{dI_{ICU}}{dt}& = k_{ICU}{\varvec{I}}_d-\frac{1}{\tau _{R_{ICU}}}I_{ICU}. \end{aligned}$$

$k_{h}$ and $k_{ICU}$ are parameters that include the percentage of active infected cases that evolve into hospitalized or ICU, respectively, and the rate at which these transitions happen. $\tau _{R_{h}}$ and $\tau _{R_{ICU}}$ are the characteristic times required for a patient to move out of the hospitalized and ICU states, respectively.

For simulation purposes, the rate of change in the total number of infections (detected and undetected) can be calculated by considering the positive flow into compartment ***I*** (see Eq. 4),

13 $$\begin{aligned} \frac{dN_T}{dt}=\frac{1}{\tau _I}{\varvec{E}}. \end{aligned}$$

### Data and model fitting

In order to perform the present study, the data sets corresponding to daily infected, daily deceased, hospitalized and ICU patients in Portugal were obtained from the portuguese Directorate-General of Health website^21^. The sets corresponding to the cumulative detected cases and cumulative deaths were also analyzed because, even though they are determined from the daily infected and daily deceased data sets, respectively, they present very different time derivatives (see Eqs. 8, 9 and Eqs. 7, 10). The simultaneous fit of instantaneous and cumulative data contributes, therefore, to the stabilization of the model fitting least squares minimization process, making it more likely to converge regardless of high frequency (daily) fluctuations.

The model fits were performed with an open-access user-friendly online platform *fitteia*® (fitteia.org) that uses the non-linear least-squares minimization method with a global minimum target, provided by the numerical routine MINUIT from the CERN library^22^.

As the compartmental models require the resolution of differential equations, the Runge-Kutta method integrated in fitteia® was used in the minimization process. After setting the values for each population compartment and parameter at initial time, $t_0$, a Runge-Kutta iteration with a time resolution fixed to 0.01 days was applied. Next, the solutions for each equation were compared with the existing data in a loop until a global least-squares minimum in the model parameters space was obtained.

### Fixed parameters and general assumptions

In view of the fact that the available data sets are not enough to make an independent estimation of all model fitting parameters, assumptions had to be made for the parameters presented in Table 1.

**Table 1:** Parameters that could not be independently estimated by model fitting the data sets corresponding to infected, dead and hospitalized.

Parameter	Value
$t _0$	day 56 - February 26
$S_0$	10.2 million people*
$I _0$	3
$\tau _I$	3.5 days
*d*	0.25 (0.15 for t$\in$ [358;399])
$\tau _{Id}$	6.25 days
*l*	0.02
$\tau _{R}$	14 days

*Total Portuguese population, which was assumed constant.

Although the first COVID-19 cases in Portugal were detected on March 2, 2020 (day 62), those patients were known to have developed symptoms as early as February 26^23^. Therefore, $t_0$ was set to 56, which is the day of year 2020 corresponding to February 26.

To accommodate for the initial growth of COVID-19 detected cases, a value of $I_0$ equal to one was insufficient. $I_0$ was, therefore, set to three, the smallest value that could explain the observed data. This indicates that on February 26 there were three infectious individuals in Portugal. The need for more than one patient zero may be related to the initial dissemination of the disease across different regions of the Portuguese territory.

The initial value for the susceptible population, $S_0$ was set to the total population of Portugal, as explained in “Model” section.

The characteristic evolution time from exposed to infectious, $\tau _I$, was set to 3.5 days. The reason for this lies in the fact that individuals become infectious about two days before becoming symptomatic. Since the incubation period for this virus is 5 days in more than 90% of the cases and outliers usually fall over this 5 day period, 3.5 seems to be a reasonable assumption^24^.

**Figure 2:**
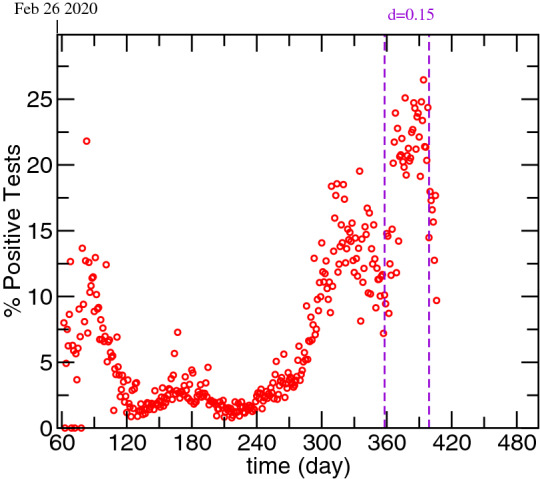
Evolution of the percentage of positive test in Portugal.

The detection ratio, *d*, and the characteristic detection time, $\tau _{Id}$ are variables that are interconnected. It is possible to obtain the same result if both parameters are multiplied or divided by the same value. Here we considered a combination of d=0.25 (25% of cases are detected) and $\tau _{Id}$=6.25 to reflect the portuguese case. As it can be observed in Fig. 2, the post-Christmas outbreak was associated with a 10% increase in the percentage of positive PCR tests. As a consequence, we reduced the fraction of detection to 0.15 for the time range associated with increased positivity (between day 358 and 399).

The fraction of people that die was calculated by dividing the most recent total number of deaths by the sum of the total number of deaths and recovered.

Portugal was one of the countries that did not provide systematic data for the recovered. This compartment is the one that is most subject to reporting delays and, if the reporting delays are not systematic, the data present several discontinuities that are difficult to incorporate in the model. We chose to attribute 14 days to this characteristic recovery time, based on what was observed for other countries^11^.

## Results

### Model fits

**Figure 3:**
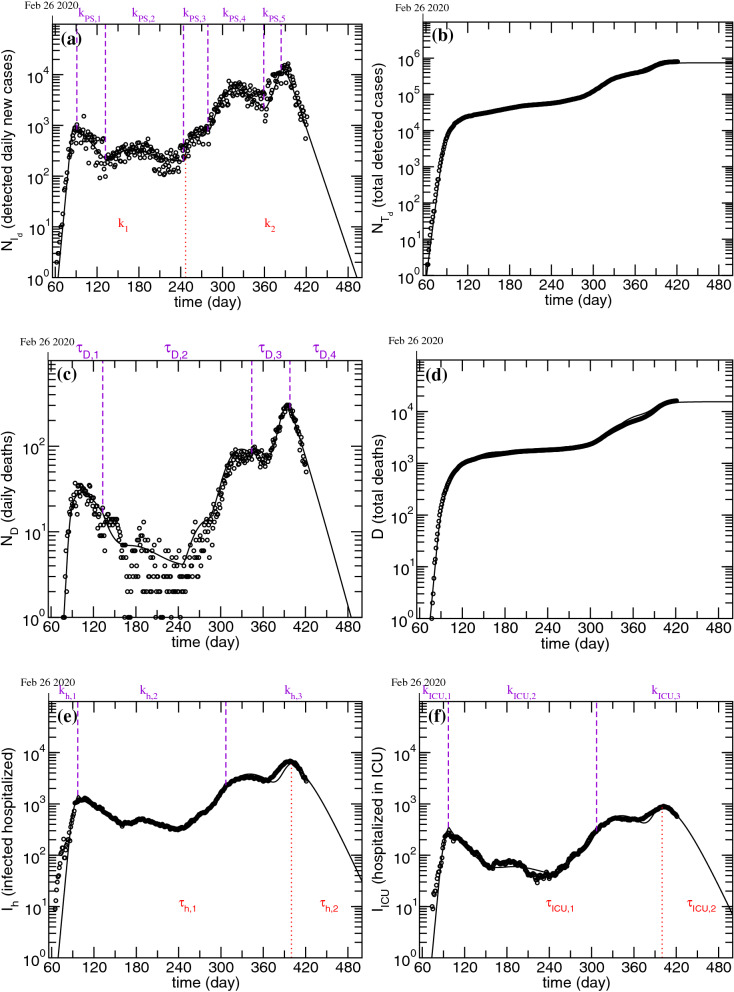
Model fitting results obtained for the application of the modified PSEIRD(S) model to the different data sets, starting from the assumptions presented in Table 1. The details regarding the auxiliary lines are explained in the text.

The results obtained from fitting the infectious, dead and hospitalized data until February 24 2021 are presented in Fig. 3 and the optimized parameters are summarized in Table 2. Figure 3a shows the time evolution of the detected daily new cases, $N_{I_d}$. The violet dashed lines show the moments when the rate $k _{PS}$ changed, while the dotted red line corresponds to the changing moment for the infection rate, *k*. In Fig. 3b are presented the cumulative number of detected infections, $N_{T_d}$. Figure 3c depicts the time evolution of the daily deceased and the violet dashed lines mark the moments when the characteristic death time, $\tau _D$, changed. In Fig. 3d it is possible to observe the time evolution of the cumulative number of deaths. Figure 3e,f show the results obtained for the hospitalized and ICU patients, respectively. In these figures, the dashed violet lines mark the changing moments for the respective hospitalization rates, $k_{h}$ and $k_{ICU}$, while the red dotted lines shows the instant when the characteristic times, $\tau _{R_{h}}$ and $\tau _{R_{ICU}}$, were changed. For all plots, the horizontal axis presents the elapsed time from January 1, 2020.

As it can be observed in Fig. 3, the simultaneous fit of the modified PSEIRD(S) model to the $N_{I_d}$, $N_{T_d}$, ${\varvec{D}}$, $N_{D}$, $I_h$ and $I_{ICU}$ compartments provides very good results, whose representation was extended in order to obtain a projected scenario, assuming that none of the parameters changes. If confinement conditions are maintained, it is possible to observe that the number of detected daily infections decreases to zero by May 15 (day 500) .

**Table 2:** Model fitting optimized parameters. Some of the parameters fitted vary discretely with time.

Parameter	Value
$k _{PS}$ (day$^{-1}$)	
$\alpha$ (day$^{-1}$)	$1.3\times 10^{-3}$
*k* (day$^{-1}$)	$0.9 {\mathop {|}\limits ^{247}} 0.3$
$\tau _D$ (day)	$5.5 {\mathop {|}\limits ^{133 }}16 {\mathop {|}\limits ^{344}} 9.6 {\mathop {|}\limits ^{398}} 50$
$k_{h}$ (day$^{-1}$)	$3.0 \times 10^{-2} {\mathop {|}\limits ^{97}} 8.5\times 10^{-3}{\mathop {|}\limits ^{307}} 5.2\times 10^{-3}$
$k_{ICU}$ (day$^{-1}$)	$6.8\times 10^{-3} {\mathop {|}\limits ^{97}} 9.9\times 10^{-4}{\mathop {|}\limits ^{307}} 7.4\times 10^{-4}$
$\tau _{R_{h}}$ (day)	$12.4 {\mathop {|}\limits ^{400}}7.2$
$\tau _{R_{ICU}}$ (day)	$13.7 {\mathop {|}\limits ^{400 }}8.9$

In that case, the value of the parameter is preceded by the day from which it applies and may be followed by the day from which it no longer applies.

As it can be observed, the modified PSEIRD(S) model explains complex disease dynamics, such as multiple outbreaks. These outbreaks are described as a flow of people from the ***P*** to the ***S*** compartment, whose magnitude increases for larger outbreaks, as seen from the different $k _{PS}$ values, presented in Table 2.

The change observed for the *k* value in the beginning of September 2020 (day 247), can be assigned to the establishment of strict measures related with extending the use of masks in outdoor public places, in order to prepare a secure reopening of services, such as schools.

For the hospitalized cases, it was not possible to make a fit considering a unique value of $k_{h}$ or $k_{ICU}$. The values were different for different outbreaks but can be estimated by the initial increase observed for the different waves of infection. The different values of $k_{h}$ and $k_{ICU}$ are consistent with the fact that the rate at which patients are admitted to hospitals is largely dependent on the age stratification related with a specific outbreak (for example, an outbreak where the elderly are the most affected should be related with a larger value of $k_{h}$ and $k_{ICU}$) and/or to the season at which it happens (winter season should increase the infected percentage and/or the rate at which patients are hospitalized). Regarding the characteristic recovery time from a hospitalization state, it was possible to make the fits assuming a single value for the different outbreaks except for the most recent one. This outbreak was the most severe in Portugal so far and lead many hospitals to exceed their capacity for receiving COVID-19 patients. Under such conditions, it is impossible to provide patients with the highest health care service quality, which leads a larger number of hospitalized individuals to die.

The characteristic death time also presents different values for different outbreaks. Naturally, when the number of hospitalized increases to values close to the limits of the health care system (less than 2000 ICU beds in Portugal^25^), mortality tends to happen at a faster rate. At the beginning of the epidemic in Portugal the characteristic death time may have had a smaller value because the knowledge of the virus and of effective therapeutics was probably insufficient at that time.

### Remaining compartments and model simulations

**Figure 4:**
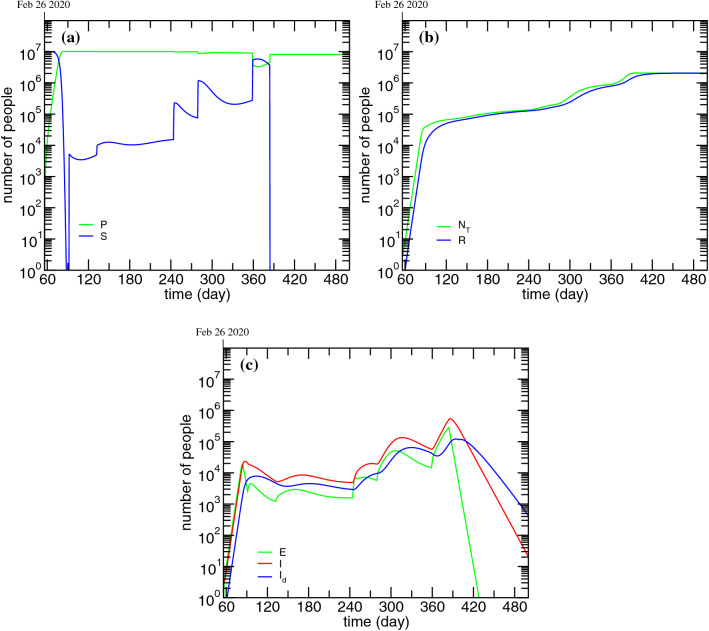
Time evolution of the total cases and of the modified PSEIRD(S) model compartments for which there is no available data or the data is not consistently reported: (**a**) ***P*** and ***S***; (**b**) NT$_d$ and ***R***; (**c**) ***E***, ***I*** and ${\varvec{I}}_d$ - c). The plots were obtained taking into account the same assumptions for the fixed parameters presented in Table 2 and the same optimized parameters presented in Table 2.

Starting from the assumptions presented in “Fixed parameters and general assumptions” section and the model fitting parameters presented in Table 2 it was possible to generate the time evolution of the compartments for which there is no available data. It is important to note that these time-series are a necessary step to solve and fit the solutions of the set of differentials equations (Eqs. 1–7), as it is not possible to determine one compartment without considering the remaining. The time evolution of the ***P***, ***S***, ***R***, ***E***, ***I*** and ${\varvec{I}}_d$ compartments, as well as NT$_d$, is presented in Fig. 4.

In Fig. 4a it is possible to see the time evolution of the ***P*** and ***S*** compartments. This figure puts into evidence the transitions between these two sates and the effective normalization of the flows. When the number of susceptible increases, the number of protected has to decrease by the same amount. Figure 4b shows the total number of detected and undetected cases and recovered. As it can be seen, in view of the fact that a small percentage of individuals dies with COVID-19, the number of recovered tends to the total number of cases minus the total deceased. In Fig. 4c is depicted the time evolution of simulated compartments ***E***, ***I*** and ${\varvec{I}}_d$. Compartment ***E*** is directly related to compartment ***I***, which directly relates to compartment ${\varvec{I}}_d$. This figure evidences the different characteristic times to move out of these three states. Clearly, the number of exposed individuals decreases much more steeply than the number of infected non-isolated and infected detected. This fact is consistent with a much faster transition out of this compartment, with a characteristic time $\tau _I$ equal to 3.5 days.

Among all the compartments, the infected non-isolated, ***I*** (Fig. 4c), is crucial for the description of recurrent outbursts, as these individuals, unaware of their infectious state, silently spread the disease. In fact, the dramatic outcome caused by allowing people to move freely on Christmas can be explained by the following simple reasoning based on this compartment. According to the obtained results, on Christmas eve there were about 58000 individuals in compartment ***I***. Considering that all of these spent Christmas with four other relatives, that means at least 232000 non-isolated infected cases by new year’s eve. This value might even be underestimated, as it does not account for the subsequent gatherings that might have taken place during this season festivities (e.g. multiple meetings with parents and in-laws, parties for exchanging gifts, etc). In view of this, it becomes clear that the number of daily cases is not enough to characterize the state of the pandemic. A closer analysis of the curves presented in Figs. 3 and 4 shows that, although the detected daily new cases drops to zero by May 15 (day 500), the number of infected non-isolated is around 20 on the same date. This number is more than sufficient to generate new outbreaks upon careless reopening.

**Figure 5:**
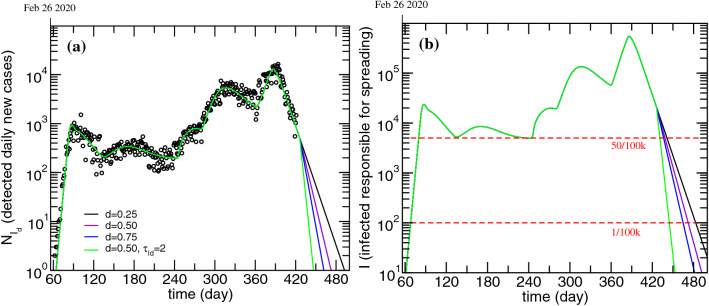
Evolution of the detected daily new cases (**a**) and infected non-isolated, responsible for spreading the disease (**b**). Effect of different values of *d* keeping $\tau _{Id}$ equal to 6.25 days and effect of increasing d to 0.5 while decreasing $\tau _{Id}$ to 2 days. The parameters were changed only after March 1.

The model fitting software - *fitteia*® - also provides a straightforward way of making simulations exploring the effects of varying any model parameter in anticipation of confinement/reopening policy changes. Therefore, simulations were made in order to understand how testing can be used to decrease the present confinement duration. For instance, the level of testing is here related with *d* and $\tau _{Id}$, so we have simulated different epidemic scenarios starting from March 1 (day 426). The results for these simulations are presented in Fig. 5 for $N_{I_d}$ and ***I***. In Table 3 are presented the dates and $N_{I_d}$ values corresponding to different ***I***-related milestones: 50/100k inhabitants and 1/100k inhabitants.

**Table 3:** Summary of 2021 dates when the silent spreaders number decreases, either to 50 per 100k or to 1 per 100k, and respective daily new cases obtained for the different scenarios projected in Fig. 5.

Scenario based on *d*=0.25 and $\tau _{Id}$=6.25	$I=50/100$k		$I=1/100$k	
	Date$^{(\text {day})}$	$N_{I_d}/ 100$k	Date$^{(\text {day})}$	$N_{I_d}/ 100$k
*d*, $\tau _{Id}$	16/Mar$^{(441)}$	1.22	26/Apr$^{(482)}$	0.027
$2\times d$, $\tau _{Id}$	13/Mar$^{(438)}$	0.99	16/Apr$^{(472)}$	0.01
$3\times d$, $\tau _{Id}$	11/Mar$^{(436)}$	0.84	8/Apr$^{(464)}$	0.007
$2\times d$, $0.33\times \tau _{Id}$	5/Mar$^{(430)}$	1.18	19/Mar$^{(444)}$	0.019

The numbers in superscript correspond to the number of days elapsed from January 1, 2020.

As it is economically unsustainable to maintain a state of “endless” confinement (e.g. until ***I*** goes to zero), it is important to evaluate the secure reopening of schools and of other services, especially in view of the dramatic decrease in detected infections and deaths that is currently being observed. As a reference we considered a value of 50/100k silent spreaders, as this was the value obtained for August 31 2020, prior to the first reopening of schools.

Looking at Fig. 5 and Table 3, it becomes clear that the larger the value of *d*, the sooner the 50/100k plateau is reached. However, upon tripling *d*, it is only possible to reach the 50/100k plateau 5 days sooner, which is a relatively small gain compared to the investment that would have to be made to target such a high detection fraction. Testing is, indeed, a crucial factor to reduce the propagation of the disease, however the characteristic time it takes for an infectious individual to receive a positive COVID-19 test result, $\tau _{Id}$, should also be taken into account. In fact, the best projected scenario corresponds to doubling the value of *d* while decreasing the characteristic detection time to two days. Although it is possible to analyze scenarios for arbitrary values of *d* and $\tau _{Id}$, in practical terms, maximizing *d* definitely has an economic cost and probably will also have a negative effect on $\tau _{Id}$ for the same testing quality. Note that, as the target plateau gets lower (e.g. 1/100k), the gains that may happen as a result of changing *d* or $\tau _{Id}$ become more significant.

**Figure 6:**
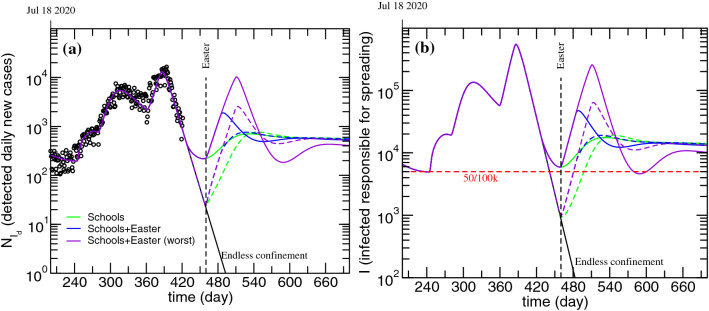
Evolution of the detected daily new cases (**a**) and infected non-isolated, responsible for spreading the disease (**b**). Impact of reopening schools on March 1 (solid lines) or April 1 (dashed lines) and the impact of doing so together with allowing Easter celebrations to different extents.

In order to test the model sensitivity to the onset of possible reopening strategies, such as reopening schools or allowing Easter celebrations to take place, the ***P***-***S*** flow associated with the largest increase of cases while schools were functioning ($k_{PS}$=1.6) was applied either from March 1 or from April 1. Furthermore, schools reopening was paired with Easter celebrations by assuming the same ***P***-***S*** flow obtained for Christmas ($k_{PS}$=27.8). Two cases were considered for Easter: either the ***P***-***S*** leakage lasts for 25 days, as was observed for Christmas, or it lasts 50 days. The results of these simulations for $N_{I_d}$ and ***I*** are presented in Fig. 6.

The model is clearly sensitive to the onset of school activities, especially when an Easter related leakage is considered. If schools are reopened in April, there is a smaller tendency for uncontrollable infections growth, as naturally expected. This fact, although intuitively trivial, is hardly ever expressed by actual numbers, which demonstrates the advantage of using a differential equations solver/fitter and compartmental models such as either the original or the modified PSEIRD(S) to project different scenarios.

It is important to stress that the magnitude of the leakage or its duration can have unpredictable values, depending on the efficacy of applied measures and on the extent of compliance. In case of doubling the time range within which the Easter leakage is active, the resulting epidemic wave reaches a much higher amplitude, as can be seen in Fig 6 (violet lines).

If we now go back to the simulation for the ***P*** compartment presented in Fig. 4a it is clear that, even taking the Christmas leakage into account, the amount of individuals that are still in this compartment is much larger than the number of people that were infected with COVID-19 in Portugal so far. This fact makes it possible to project future epidemic scenarios that are much worse than the one observed immediately after Christmas. The way to dramatically reduce this risk is to vaccinate as many individuals as possible to deplete the ***P*** compartment of people and reduce its capacity to feed the susceptible compartment.

**Figure 7:**
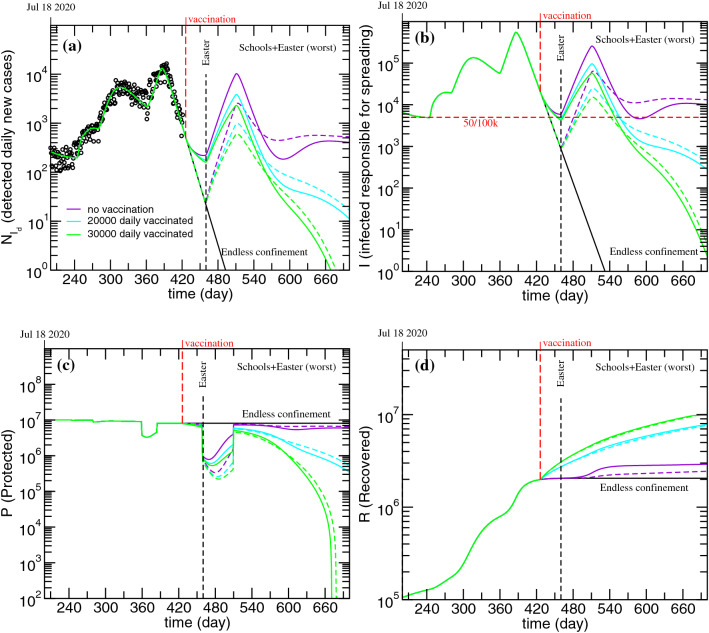
Evolution of the (**a**) detected daily new cases; (**b**) infected non-isolated, responsible for spreading the disease; (**c**) protected and (**d**) Recovered. Worst case presented in Fig. 6 and the effects of different vaccination strategies. Solid lines-schools reopen on March 1; Dashed lines-schools reopen on April 1.

In Fig. 7 we present a few projections to explore the effect of different vaccination rates starting from the worst scenarios presented in Fig. 6 (violet curves). Since vaccination related scenarios are being projected, we have also included the simulations of the ***P*** and ***R*** compartments, which are the ones directly affected by the vaccination flow. In the simulations presented in Fig. 7 it was assumed that vaccination started from March 1 with different numbers of effective daily vaccinated. In view of the fact that some vaccines require two doses to fully immunize an individual, to achieve the effective daily vaccinated number considered for the simulations in Fig. 6, the number of vaccine doses administered per day has to be adapted according to the type of vaccine.

As it can be observed in Fig. 6, if the number of effective daily vaccinated is 30000 (60000 vaccine doses for two-dose vaccines), then it is possible to decrease the number of infectious cases to zero before the end of 2021. Note that, as it can be observed in Fig. 7c,d, as the number of daily vaccinated individuals increases, the number of individuals in the ***P*** compartment decreases, while the number of recovered increases.

**Figure 8:**
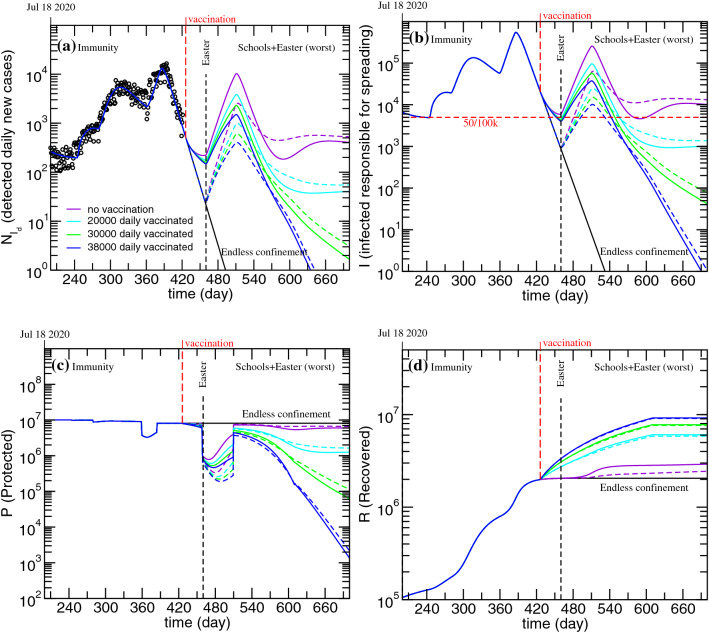
Evolution of the detected daily new cases (**a**), infected non-isolated, responsible for spreading the disease (**b**), protected (**c**) and Recovered (**d**). Worst case presented in Fig. 6 and the effects of different vaccination strategies considering that vaccine related immunity lasts five months. Solid lines-schools reopen on March 1; Dashed lines-schools reopen on April 1.

It is also possible to account for vaccination related immunity loss. For example, assuming that immunity lasts five months, five months from March 1, the number of individuals that will have lost immunity is equal to the number of daily vaccinated. In Fig. 8, it is possible to see the simulations based again on the worst scenario presented in Fig. 6 and where the effects of different daily vaccinated, paired with a five months lasting immunity, are described.

As immunity loss is considered, the daily vaccinated needed to eliminate COVID-19 infectious cases by the end of 2021 increases from 30,000 to 38,000. In this case, by the end of 2021, the number of protected individuals does not decrease as much as when immunity loss was not considered (see Figs. 7c, 8c), as one may have expected.

## Conclusion

In this work we have presented the analysis of the COVID-19 epidemic data for Portugal using the modified PSEIRD(S) model and an open-access online platform - *fitteia*® at fitteia.org - that, among other functionalities, enables the fitting of differential equation solutions to different sets of epidemiological data. The simultaneous analysis of the infected, deceased and hospitalized data sets, along with considering both daily and cumulative data, facilitated the process of finding the set of parameters corresponding to the global least-squares minimum. Furthermore, considering the data from the beginning of the epidemic in Portugal has put into evidence similarities, differences and variability of model parameter for the successive outbursts.

The modified PSEIRD(S) model includes some aspects of social behavior and is, therefore, particularly interesting for projecting different scenarios by changing parameters that can be translated into specific actions, such as increasing the fraction of detected cases, decreasing the characteristic detection time and analyzing different numbers of daily vaccinated individuals. The attempt to predict any future evolution of the pandemic is prone to failures, as human and social behavior are essentially unpredictable. The different scenarios presented in this work illustrate how the time series of the different population compartments will evolve assuming that the general response to reopening will follow the same trends observed so far. Naturally, much worse scenarios could be evaluated, but we expect that this work will contribute to put into evidence how to avoid past mistakes.

Regarding the model and the data analysis methods followed, we expect to have made clear that incorporating new compartments, parameters and assumptions (ex: different variants of the virus or age stratification) will be within reach with a relatively small effort, taking into account the technical possibilities the open-access platform at fitteia.org available for general use.

We trust that both the method and the analytical possibilities offered by *fitteia*® could provide a positive contribution for the analysis of the current pandemic in other countries and of other epidemics. This tool provides a user-friendly way of fitting compartmental models to epidemiological data and, therefore, enables the user to make more robust projections starting from the description of past observations. In fact, if a model does not explain the details of the past, there is a good chance that it will not be appropriate to explore the near future.

## Data Availability

All data is available in the main text or is from public repositories. Fitteia templates will be available online at https://github.com/fitteia/COFIT.

## Code availability

Relevant code and fitting results are available online at https://github.com/fitteia/COFIT.
